# Who seeks help in a crisis? Temporal analysis of anti-trafficking helpline contacts over the pandemic

**DOI:** 10.1186/s12889-025-22312-3

**Published:** 2025-04-05

**Authors:** Lisa Tompson, Ella Cockbain

**Affiliations:** 1 Te Puna Haumaru New Zealand Institute for Security and Crime Science, University of Waikato, Hamilton, 3240 New Zealand; 2 Department of Security and Crime Science, University College London, 35 Tavistock Square, London, WC1H 9EZ United Kingdom

**Keywords:** Migration, Violence, Domestic servitude, Forced labour, Crisis hotline, Complex systems

## Abstract

**Background:**

Human trafficking and extreme exploitation ("modern slavery") violate fundamental human rights and cause severe harm to individuals' well-being. Anti-trafficking helplines provide critical support for victims/survivors, yet little research has explored their usage patterns, particularly during crises. This study examines how the Covid-19 pandemic influenced reporting to the UK’s Modern Slavery & Exploitation Helpline, aiming to identify trends in help-seeking behaviour during this period of significant social disruption.

**Methods:**

This study analysed 8,386 cases from the Helpline between October 2016 and December 2021. We took a descriptive approach, as assumptions for causal analyses could not be met. We used seasonal decomposition methods to separate underlying trends over the pandemic from seasonal effects, focusing on caller proximity and exploitation sub-types.

**Results:**

Helpline contacts decreased following the introduction of Covid-19 restrictions, although case volume remained steady compared to pre-pandemic levels. Reporting patterns shifted: reports of criminal and sexual exploitation increased, while those of labour exploitation declined. Self-reports from victims/survivors rose notably during in-person work restrictions, particularly for labour exploitation in essential industries. Reports from the public about suspicious activity dropped and did not rebound post-lockdown, suggesting long-term changes in public reporting behaviour.

**Conclusions:**

The study demonstrates how the Covid-19 pandemic affected reporting to a major anti-trafficking helpline, revealing increased self-reports from victim/survivors but a decline in community-based reporting. These findings highlight the importance of helplines as a public health intervention during crises and underscore the need for enhanced support infrastructure during periods of social and economic disruption. Future research should investigate the underlying causes of these shifts in reporting and explore ways to improve service access for victim/survivors of trafficking during emergencies.

## Background

Human trafficking and extreme exploitation (often termed 'modern slavery') cover multiple and varied forms of abuse [1], violate fundamental human rights [2] and can have numerous negative impacts on health and well-being [3–5]. Although generally thought to happen on a large scale, there are few robust localised prevalence estimates and global ones are notoriously contentious [6].

Victims/survivors often face intersecting marginalisations (e.g., migration status, gender, sexual identity, race/ethnicity) and can be difficult to access from a health perspective [7]. Significant global resources have focused on criminal justice responses [8], but few traffickers have been convicted in the UK or elsewhere. Consequently, attention is shifting toward a public health approach focused on prevention and harm reduction [4, 9–11].

Helplines are a key component of anti-trafficking efforts internationally [8], offering free, confidential advice and referrals to support services. Despite their potential as a pillar of harm reduction, anti-trafficking helplines have received little research attention [8]. Limited knowledge exists about what drives victims/survivors, their associates, or bystanders to seek help or report concerns. This paper explores how the Covid-19 pandemic affected contacts to the United Kingdom’s (UK) Modern Slavery & Exploitation Helpline, aiming to identify reporting trends for suspected exploitation and understand changes in help-seeking behaviour. This foundational study into anti-trafficking helpline usage in a period of significant social and economic rupture could inform future disaster planning for pandemics, natural disasters, and conflicts.

In March 2020, the UK entered lockdown to curb the spread of Covid-19, suppressing commercial activity and drastically altering daily life through stay-at-home orders. Lockdowns are a well-documented ‘exogenous socio-behavioural pattern shock’ exhibiting myriad effects on behaviour [12]. By restricting mobility, lockdowns disrupted crime 'opportunity structures', that is, the set of conditions that make an outcome more or less likely; reducing many crime types [13, 14] and increasing others, e.g. domestic violence [15]. Police had to quickly adapt to enforcing public health measures in many countries. The increased presence of people at home enhanced residential guardianship—passive surveillance by the everyday public (hereafter called residential monitoring) [16]. The opportunity structures for the occurrence, identification and reporting of trafficking were likely differentially affected depending on the exploitation type and context in question. For example, increased residential monitoring plausibly boosted opportunities to identify domestic servitude or criminal exploitation [17–19], but likely impacted little on (say) the visibility of labour exploitation in farms or factories.

Trafficking and ‘modern slavery’ are umbrella terms for a complex and vaguely defined set of issues that vary widely in context, impact, and response needs [11, 20]. This paper focuses on known or suspected cases of trafficking and other ‘modern slavery’, which we refer to hereafter as ‘exploitation’ because ‘modern slavery’ is a problematic term [8]. In the UK, the main classifications for such exploitation are criminal, labour, sexual, and domestic servitude, and individuals may be exploited in multiple ways.

Substantive variation has been identified in data on trafficking victims/survivors, leading many to advocate for disaggregation by exploitation types [20–22]. However, even within broad exploitation categories, significant heterogeneity remains. For example, criminal exploitation includes contexts ranging from cannabis farms to shoplifting, drug distribution, and benefit fraud. Similarly, labour and sexual exploitation occur across various sectors and contexts.

Despite speculation about the pandemic’s impact on trafficking, there has been little empirical research directly estimating trafficking prevalence [23], with most studies relying on proxy variables [24] due to measurement difficulties. Papers published during the pandemic predicted an increase in trafficking occurrence, alongside greater difficulty in detection [7, 25]. Official data suggests the latter may be true [26], although existing data may obscure conflicting trends in trafficking prevalence and help-seeking behaviour.

The effects of pandemic restrictions on different markets—both licit and illicit— varied, ostensibly affecting trafficking differently across sectors. While many labour markets stalled, essential industries (e.g., agriculture, construction) and drug markets remained active in many countries [17, 19]. Consequently, opportunities for trafficking persisted, even as operating contexts shifted significantly.

Anti-trafficking professionals rapidly raised concerns about workplace safety for exploited individuals during the pandemic, particularly regarding inadequate protective equipment and social distancing [23, 27]. Professionals further reported traffickers abandoning their victim/survivors on the streets as demand for labour fell away during lockdowns, but also stressed that some victim/survivors were confined in increasingly controlled and violent conditions ‘in private homes, factories, construction sites and other locations’ [23]. Additionally, it was suggested that confinement of domestic servitude victims/survivors with their exploiters might exacerbate abuse [28]. This heightened abuse may have overwhelmed victims’/survivors' coping mechanisms, making self-identification and help-seeking more likely [29].

The sex industry was severely disrupted by Covid-19 due to infection fears, physical distancing measures, stay-at-home orders, and increased police enforcement against street workers [30, 31]. Many consensual sex workers shifted to online platforms, such as camming and selling explicit images [32]. Similar shifts may have occurred for those being trafficked. At other times, they may have been forced to continue face-to-face services under increasingly dangerous circumstances, in turn feasibly affecting help-seeking behaviour.

Trafficking related to drug distribution^1^ adapted to pandemic conditions. In Brisbane, Australia, increased detection of drug possession and dealing during pandemic restrictions suggests that reduced foot traffic made drug market participants more visible and vulnerable to generalised policing [17]. Similarly, British police reported greater ease in identifying people running drugs during the first lockdown [18], with one officer stating it was like “shelling peas” [33]. However, exploiters quickly adapted their tactics [19]. Overall, these examples highlight the variety of mechanisms at work, the importance of context and disaggregation, and that traffickers can adjust tactics as opportunity structures shift.

Reporting behaviour to unofficial bodies, such as helplines, is influenced by two main drivers: actual exploitation and factors affecting the likelihood of reporting. Influences on the former include economic conditions, migration policies and labour market regulation [11]. Reporting is likely impacted by political attention, enforcement actions, media coverage of arrests, public awareness campaigns, and even plotlines in popular television shows [8, 34–36]. What people suspect to be’modern slavery’ may or may not correspond to legal definitions or how those affected understand their experiences. While reports are an imperfect reflection of actual exploitation, they nevertheless offer insights into how helplines are used, which groups report, and which issues they highlight.

The opportunity structures (i.e., conditions) for third-party reporters encountering suspected exploitation depend on their social contacts, routine activities and mobility patterns. The chance of witnessing suspicious activity or encountering victims/survivors is influenced by these patterns, which were significantly disrupted by pandemic-related stay-at-home orders. This may explain why Polaris, the US trafficking helpline, reported changes in the composition and relationships of callers during the pandemic, despite no significant change in overall case volume [37].

Having reviewed the theorised and empirical patterns seen over the pandemic for different types of exploitation, we distil our predictions about helpline reporting behaviour into two groups: mechanisms affecting identification and reporting and those influencing actual exploitation. Our hypotheses are:

### Primarily reporting-driven

Due to restricted mobility during stay-at-home orders:

- 1. Reports from the public about suspicious behaviour will sharply decline and remain low until orders are lifted.^2^

Due to increased residential monitoring during stay-at-home orders:

- 2. Reports of domestic servitude will increase.
- 3. Reports of criminal exploitation will increase.

### Primarily exploitation-driven

Due to working conditions becoming more unsafe during in-person work restrictions^3^:

- 4. Self-reports from potential victims/survivors will rise.

Due to a contracted market for in-person sexual services during in-person work restrictions:

- 5. Reports of in-person sexual exploitation will decrease.

Due to changes in service delivery during in-person work restrictions:

- 6. Reports of online sexual exploitation will increase.

Due to the closure of certain industries, reports of potential labour exploitation cases during in-person work restrictions:

- 7. Will be suppressed in non-essential public-facing industries.
- 8. Will remain stable in essential industries^4^ (e.g., construction, agriculture).

These two groups of hypotheses may point in different directions, potentially obscuring trends. Additionally, the mechanisms may overlap, as reflected in H_4–6_.

## Methods

### Sample

In a field where data access is a common barrier to empirical research, we capitalised on rare, independent access to a large dataset. Unseen, the NGO that operates the Modern Slavery & Exploitation Helpline (hereafter, 'the Helpline'), provided two tranches of anonymised data on cases classified as potential ‘modern slavery’ (i.e. trafficking and other extreme exploitation) (*n* = 8,386). The data period was 10 October 2016, to 31 December 2021.^5^ All data were anonymised, and participant confidentiality and data protection standards were upheld throughout. The study was approved by the UCL Research Ethics Committee (reference: 5160/002).

The data consist of self-selected contacts to the Helpline via phone, email, web form, or app, primarily from the UK, though contacts may originate from any country. Trained call handlers are available 24/7 and follow a protocol for safety checks. Activity assessed as potential (extreme) exploitation is classified as a 'case'. Unseen reports using a victim-centred approach, not seeking to verify or investigate information provided: the caveat ‘potential’ applies as reports cover both known and suspected but unconfirmed exploitation. Where appropriate, contacts are referred to support agencies.

The dataset covers just over five years of potential exploitation cases. For each case, information on the nature of exploitation, the caller type (e.g., organisation), and 'caller proximity'—how closely the caller knew the potential victim—was included. Caller proximity is categorised into four types: a) observation of suspicious activity, b) indirect contact with the potential victim,^6^ c) direct contact with the victim, and d) self-reporting by the victim. A detailed description of data structure, quality assurance, and data manipulation is provided elsewhere [8]. We include a summary of key variables by exploitation subtype in Table 1.

**Table 1:** Cases (*n* = 8,386) by exploitation type, sector and sub-sectors, and caller proximity

			**CALLER PROXIMITY**					
**Exploitation category**	**Exploitation sector category**	**Exploitation sector sub-category**	**Victim self-report**	**Direct contact with PV**	**Indirect contact with PV**	**Observation of suspicious activity**	**Unknown**	**Total**
Labour exploitation	Care sector	-	11	25	8	8	1	53
	Construction	-	43	149	41	275	12	520
	Entertainment	-	5	12	1	3		21
	Food production	Agriculture / farm	64	45	17	45	2	173
		Factory	5	6	3	5		19
	Hospitality	Hotel / Motel	10	17	3	8	3	41
		Take away / Restaurant	38	105	30	75	8	256
		Other	10	24	4	21	1	60
	Manufacturing	Factory	22	34	9	13	2	80
		Other	8	10	4	8		30
	Maritime/boat/shipping	-	3	10	4	4		21
	Services	Beauty / Spa	5	145	21	197	6	374
		Car wash	28	257	69	703	21	1,078
		Shop	4	13	3	8	1	29
		Other	12	69	14	33	6	134
	Transportation	-	14	22	8	13		57
	Various	-	52	82	22	31	5	192
	Other	-	83	180	44	124	10	441
**Subtotal**			**417**	**1,205**	**305**	**1,574**	**78**	**3,579**
Sexual exploitation	Commercial	Brothel	137	273	70	146	20	646
		Hotel / Motel	10	11	2	8		31
		Online	7	26	5	21	26	60
		Private Home	10	57	8	53	57	133
		Street	1	3	2	10	3	16
		Various	9	11	3	5	11	28
	Various	-	17	95	18	37	9	176
	Other	-	29	97	13	45	1	185
**Subtotal**			**220**	**573**	**121**	**325**	**36**	**1,275**
Unknown	Other	-	272	1,042	161	137	135	1,747
**Subtotal**			**272**	**1,042**	**161**	**137**	**135**	**1,747**
Domestic servitude	Domestic work / au pair / nanny	-	137	365	75	98	15	690
**Subtotal**			**137**	**365**	**75**	**98**	**15**	**690**
Criminal exploitation	Criminal	Benefit fraud	2	8	4	1		15
		Cannabis farm	25	76	19	7	2	129
		Other drugs	63	210	33	12	4	322
		Pickpocketing		1		1		2
		Shoplifting	4	11	7	4		26
		Street begging	3	63	12	121	1	200
	Other	-	2	9	4			13
	Various	-		4	1			5
**Subtotal**			**116**	**456**	**89**	**150**	**10**	**821**
Various	Various	-	70	119	26	52	7	274
**Subtotal**			**70**	**119**	**26**	**52**	**7**	**274**
**OVERALL TOTAL**			**1,232**	**3,760**	**777**	**2,336**	**281**	**8,386**

#### Data preprocessing

Data manipulation and analysis were conducted in R [38]. Contact (caller^7^) information was merged with case data (a many-to-one relationship), resulting in a dataset where each row represents a case. The mean number of contacts per case was 2.3 (range: 1–73) and the mean time between the first and last contact was 12 days (range 0–1,658 days). All variables used in the analysis were sourced from the 'case' data file, which reflected the most up-to-date information available (e.g., any changes in details were updated accordingly). In cases with multiple contacts spanning different caller proximity categories, the analysis used the category with the closest proximity.

The data were ordered chronologically into a time series. According to the Oxford COVID-19 Government Response Tracker [39], the first UK public restrictions were imposed on March 13, 2020, leaving 41 months (1,249 days) before restrictions and 22 months (444 days) after. The resulting time series contains 63 data points. Cases were classified as ‘before restrictions’ if the earliest and latest contacts per case fell before March 13, 2020, and ‘during restrictions’ if both followed this date. Cases that spanned both periods were excluded (*n* = 113, 1.3% of cases). While restrictions varied in intensity and UK region in the ‘during’ period,^8^ systematic application of these variations to individual cases was unfeasible, as cases could involve cross-border exploitation. Furthermore, population compliance with restrictions likely reduced over time, undermining temporal consistency [40].

#### Analysis

Due to the heterogeneous non-stationary nature of our data, lack of suitable control groups, and small sample sizes in key subsets, we could not use interrupted time-series, difference-in-difference techniques, or segmented regression due to unmet assumptions [41–44]. Given that trafficking and exploitation are characterised as complex systems with numerous unobserved confounders affecting reporting behaviour [8, 10, 45], rival hypotheses for trends in Helpline contacts could not be discounted. Accordingly, we opted for context-sensitive descriptive analysis as an early step in understanding changes in contacts over the pandemic. This approach aims to observe developmental trends in Helpline contacts, rather than make causal inferences [42].

In what follows, we present a series of observational time-series charts. Seasonal decomposition [46] was used to parse underlying trends from seasonal effects and random noise. We report both raw time-series data and the extracted trends. Additionally, we comment on proportions of different caller proximity types before and during the pandemic, but caveat these are exploratory, not explanatory analyses. We do not use t-tests or chi-square tests, as they ignore underlying time-series trends and could yield spurious effects. Throughout the study, we consulted with Helpline staff to clarify data- and process-related queries.

## Results

In the six months following the introduction of Covid-19 restrictions in the UK, contacts to the Helpline decreased, although the number of cases arising from those contacts was consistent with the same period in 2019 [27]. However, the nature of cases shifted, with reports of labour exploitation decreasing and of criminal and sexual exploitation increasing. During periods of the strictest restrictions, Helpline contacts dropped [47].

**Fig. 1:**
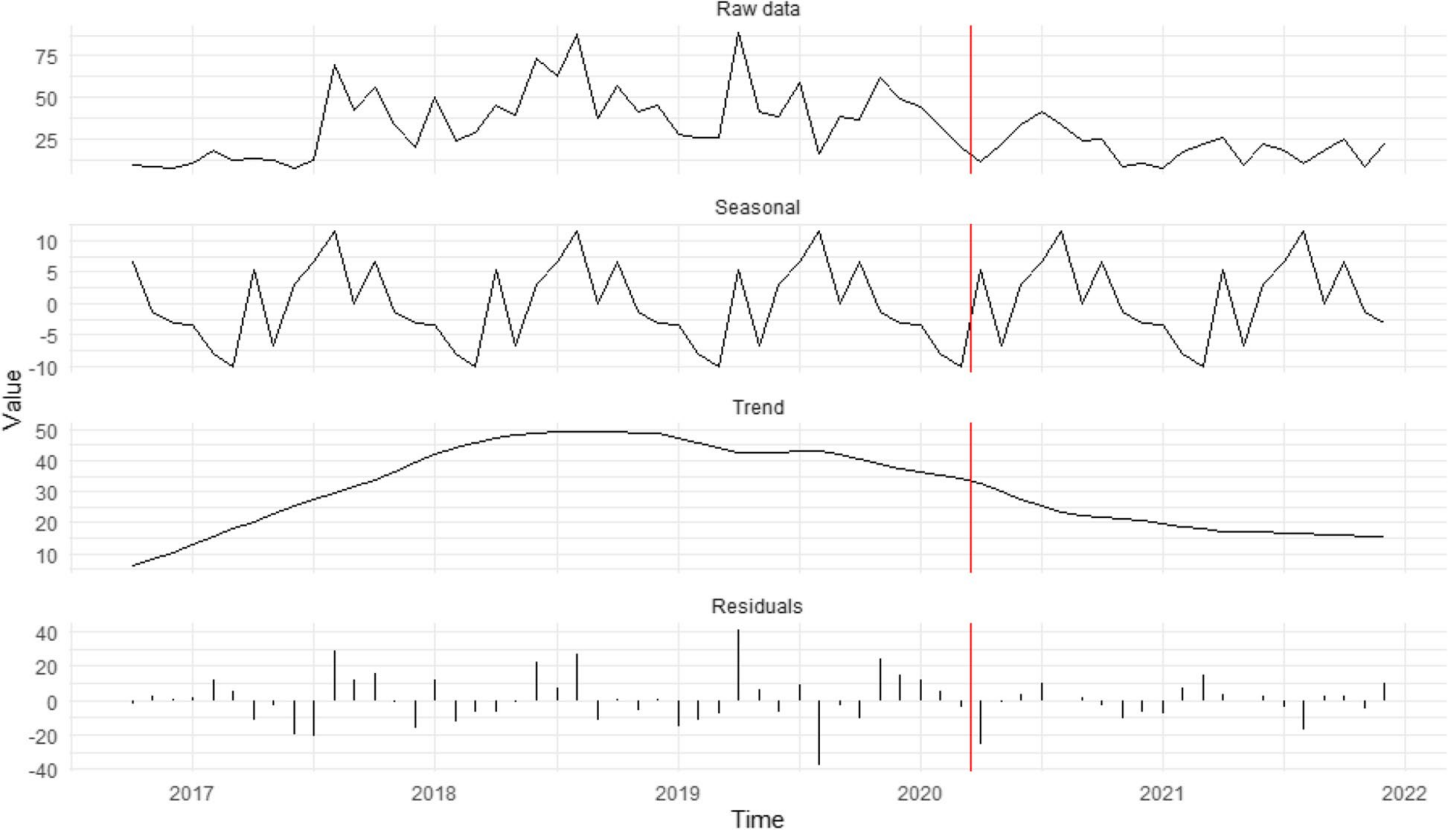
Seasonal decomposition time-series of potential exploitation cases stemming from reports from the public about suspicious activity (*n* = 1,973). The red vertical line represents the introduction of restrictions

Figure 1 presents seasonal decomposition for cases opened solely due to public observations of suspicious activity. The top panel shows the overall monthly time-series, followed by the seasonal component, the trendline, and the random error terms (residuals) in subsequent panels. Figure 1 reveals that cases continued to decline after the introduction of stay-at-home orders, with minor peaks in August 2020 and May 2021 that, interestingly, did not align with official easing of restrictions [39]. Thus, H_1_, which predicted that stay-at-home orders would suppress public reports, is partially supported: contacts from the public dropped after the pandemic began. However, these contacts did not recover when stay-at-home orders were lifted and have yet to return to pre-pandemic levels, indicating the presence of unobserved confounding factors.

**Fig. 2:**
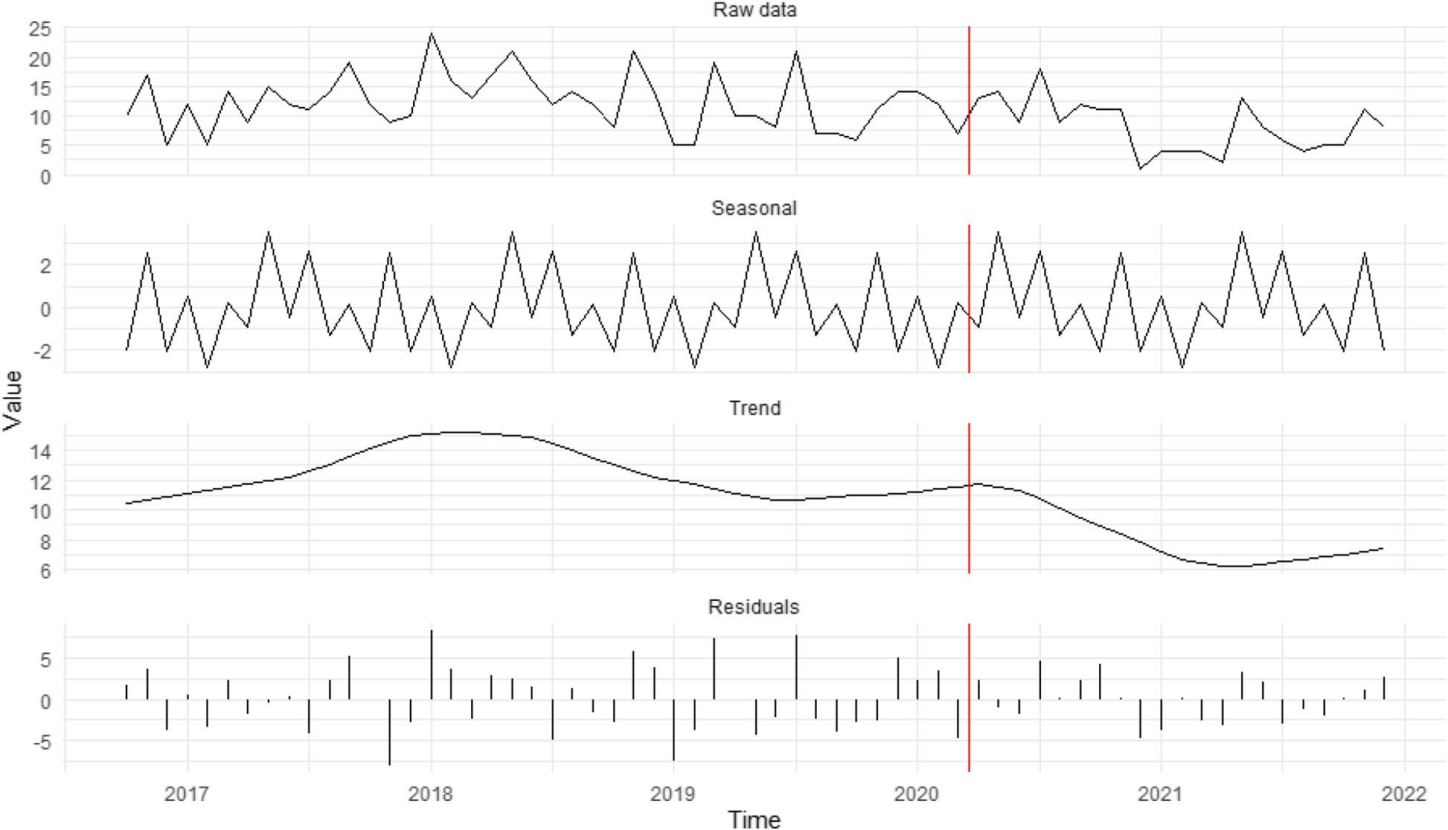
Seasonal decomposition time-series of potential domestic servitude cases (*n* = 690). The red vertical line represents the introduction of restrictions

Figure 2 shows that domestic servitude cases remained flat in the first months after restrictions were implemented, then declined noticeably in the second half of 2020. Thus, H_2_, which predicted an increase in domestic servitude cases due to greater residential monitoring, is not supported. A modest rise in potential domestic servitude cases occurred towards the end of 2021, long after stay-at-home orders were lifted. Victim/survivor self-reports were the only caller proximity type to increase for domestic servitude during the pandemic (16.2% to 28.8%), while all other types decreased.

**Fig. 3:**
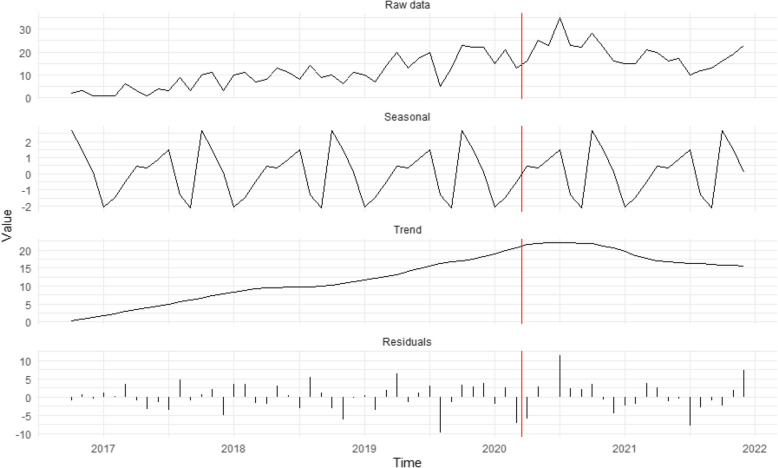
Seasonal decomposition time series of potential criminal exploitation cases (*n* = 821). The red vertical line represents the introduction of restrictions

Figure 3 shows that the increasing trend in potential criminal exploitation cases since 2017 continued after stay-at-home orders, supporting H_3_ that criminal exploitation would increase. However, unique trends in caller proximity suggest that residential monitoring was likely not the main driver. Observations of suspicious activity dropped from 27.8% pre-restrictions to 8.8% post-restrictions (supporting H_1_ that community reports about suspicions would decline). Self-reports by potential victim/survivors rose from 9.2% to 18.5%, and reports from people close to potential victim/survivors increased from 50.6% to 61.0%. Police contacts remained stable (15.4% vs. 16.8%), peaking in July 2020.

Reports of minors as potential criminal exploitation victims/survivors increased in 2020 (*n* = 56) compared to previous years (2017 = 13, 2018 = 17, 2019 = 41) but declined in 2021 (*n* = 33). However, small sample sizes and incomplete age data (about a quarter of cases lack coarse information on whether a minor was potentially victimised) mean these figures should be interpreted cautiously. Cases involving potential exploitation^9^ in drug distribution rose from 30.0% (*n* = 121) to 48.5% (*n* = 199) during the pandemic. The main sources of these reports were Local Authorities^10^ (*n* = 50, with 29 involving minors), potential victims/survivors (*n* = 37), and legal professionals (*n* = 28).

**Fig. 4:**
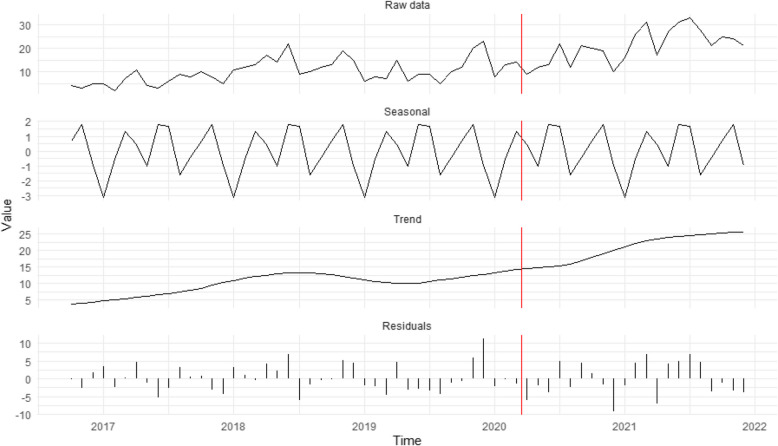
Seasonal decomposition time-series of potential exploitation cases stemming from victims/survivors self-reporting (*n* = 860). The red vertical line represents the introduction of restrictions

Figure 4 supports H_4_, which predicted an increase in self-reported cases from potential victims/survivors during in-person work restrictions. The rise was most notable in potential labour exploitation cases, increasing from 7.4% of all contacts pre-restrictions to 23.5% post-restrictions. Noteworthy increases were also seen in potential criminal exploitation (9.2% to 18.5%) and domestic servitude (16.2% to 20.0%) contacts. The increase in self-reports for potential sexual exploitation cases was more modest (15.1% to 20.0%).

**Fig. 5:**
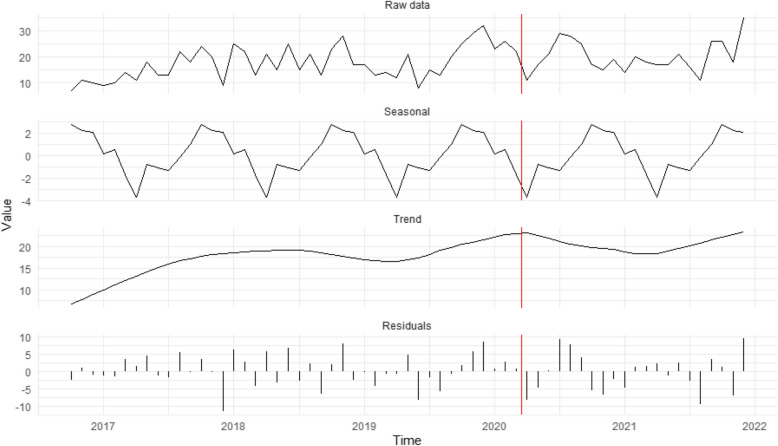
Seasonal decomposition time-series of potential in-person sexual exploitation cases (*n* = 1,158). The red vertical line represents the introduction of restrictions

Figure 5 shows that potential in-person sexual exploitation cases fluctuated after restrictions were implemented, with an increase in July and August 2020 followed by a decline over the next 12 months. Cases began to rise again in late 2021. Concerns about sexual exploitation shifting online during the pandemic were not reflected in the Helpline data. Although the proportion of potential online sexual exploitation cases increased from 7% (*n* = 53) to 12.7% (*n* = 63) after restrictions were introduced, the numbers were too small and the time-series too volatile to draw firm conclusions. Therefore, H_5_, predicting a decrease in in-person sexual exploitation cases, and H_6_, predicting an increase in online cases, are not supported.

The pandemic trends in caller proximity for potential sexual exploitation were notable. Observations of suspicious activity, mostly by the public, increased from 21.0% to 32.9% of all contacts, with small peaks in Summer 2020 and Spring 2021. These did not align with the easing of social restrictions,^11^ suggesting residential monitoring may have played a role. Cases from people in direct contact with potential victims/survivors rose slightly from a 2019 low but declined overall during the pandemic, from 49.1% to 39.5%. People in indirect contact with potential victims/survivors decreased from 11.7% to 5.4%. Self-reports from potential victims/survivors initially dropped but rose after August 2020, comprising 20.0% of cases post-restrictions, compared to 15.1% pre-restrictions. Cases involving suspected minors increased in 2020 (*n* = 47) compared to previous years (2017 = 35, 2018 = 28, 2019 = 28) but fell again in 2021 (*n* = 17).

Overall, potential labour exploitation cases declined during the pandemic. When disaggregated by caller proximity and industry, clearer trends emerge. Public observations of ‘suspicious activity’ decreased from 47.0% to 33.1% post-restrictions, offset by an increase in self-reports, which rose from 7.4% to 25.3%. Self-reports peaked in February 2021, with a secondary peak in May 2021, before declining. The most frequently reported industries for self-reports were farms (*n* = 49), construction (*n* = 17), and factories (*n* = 16)—all exempt from in-person work restrictions, lending support to H_4_ that self-reports from potential victims would increase due to more unsafe working conditions.

**Fig. 6:**
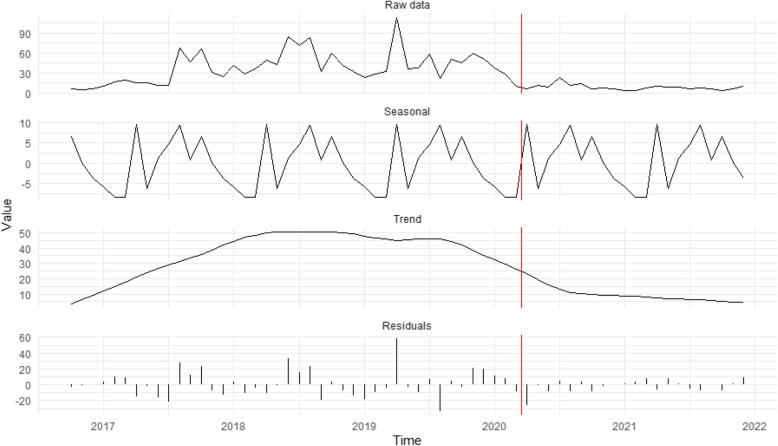
Seasonal decomposition time-series of potential labour exploitation cases in public facing non-essential industries (*n* = 1,778). The red vertical line represents the introduction of restrictions

Exploitation in public-facing non-essential industries like hospitality, beauty services, and car washes saw a sustained drop during the pandemic (Fig. 6), continuing a trend that began in mid-2019 [48]. No uptick in reporting was observed after restrictions were lifted, hence H_7_ that predicted labour exploitation cases would be suppressed during restrictions is only partially supported because no rebound in cases was observed when restrictions were lifted.

**Fig. 7:**
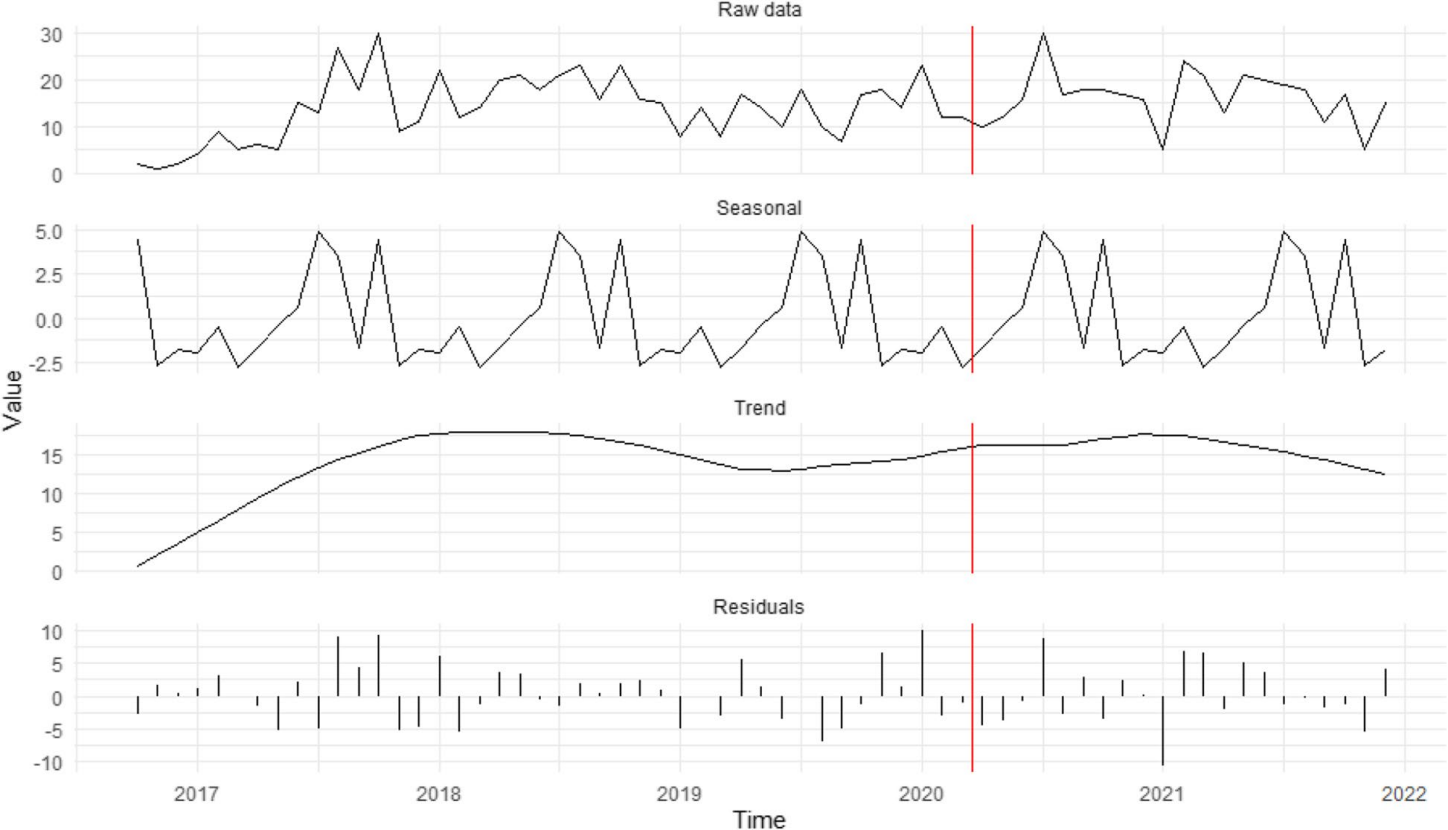
Seasonal decomposition time-series of potential labour exploitation cases in essential industries (*n* = 923). The red vertical line represents the introduction of restrictions

In contrast, potential exploitation in essential industries—care, construction, factories, farms, maritime, and transportation—continued to rise after restrictions were introduced in 2020 (Fig. 7), with a slight decrease in 2021. In addition to an increase in self-reports during the pandemic for this exploitation subtype, there were also increases in observation of suspicious activity and people with direct contact with the potential victim. Thus, H_8_, predicting stable trends in essential industries, is not supported.

## Discussion

Anti-trafficking helplines are conspicuously under-researched [8]. This study offers a descriptive analysis of how contacts to the UK’s Modern Slavery & Exploitation Helpline changed in the wake of public health restrictions, imposed to curb the spread of Covid-19. These restrictions represent a significant exogenous shock to daily routines, altering the opportunity structure (i.e., conditions) for noticing and reporting suspected exploitation and for exploitation itself occurring. Our study meets the need outlined by the United Nations for primary research into how the pandemic affected human trafficking [23]. Gaining insight into the usage of anti-trafficking helplines during crisis periods—particularly by those seeking help as victims/survivors—can shape future planning for pandemics, natural disasters and conflicts.

The findings reveal that, aligned with H_4_, self-reports from potential victims/survivors increased across all exploitation types during in-person work restrictions, with labour exploitation cases showing the most significant rise. As the primary target population, this increase is encouraging, providing insights into victims’/survivors’ needs and enabling the collection of higher quality information about the exploitation [36]. Similar self-reporting trends were noted by a US anti-trafficking helpline [37]. Although neither helpline dataset could explain this rise, our disaggregation of exploitation into essential and non-essential sectors supports the hypothesis that worsening working conditions during in-person restrictions may have played a role (H_4_).

Contacts from community members dropped significantly after restrictions were introduced. While reduced mobility due to stay-at-home orders likely limited opportunities to witness suspicious behaviour outside residential areas, this alone does not explain why community contacts did not rebound once restrictions were lifted. Additional factors may include public preoccupation with the pandemic’s health and economic impacts and a shifting political narrative increasingly focusing on ‘stop the boats’ over support for trafficked people [49, 50]. Moreover, awareness campaigns promoting the 'spotting the signs' discourse, a staple of anti-trafficking efforts, have drawn criticism for harming marginalised groups through citizen surveillance [51], and relying on ‘indicators’ of questionable utility and validity [52]. Thus, the decline in community contacts is not necessarily negative if, on balance, they cause less good than harm. That though is an empirical question that remains to be answered [8].

Another key finding was the increase in contacts related to potential criminal exploitation after restrictions were introduced. Contrary to H_3_, that proposed this would be due to residential monitoring, this rise was driven by reports from potential victims/survivors and those close to them, not community members [16], which is positive for the reasons discussed earlier. In 2020, reports involving minors may have come from adults noticing breaches of stay-at-home orders or increased pressure on exploited children to adapt their activities to avoid police attention [19].

Consistent with US Polaris data [37], Helpline contacts about potential labour exploitation in public-facing non-essential industries declined after restrictions prohibited work in these sectors (partially supporting H_7_). However, this trend began in 2019, so it cannot be fully attributed to pandemic-related restrictions. Moreover, cases did not increase after restrictions were lifted, suggesting other factors are at play, possibly related to reduced public concern about trafficking, particularly in sectors that previously received intense attention, like car washes [8]. Meanwhile, refuting H_8_, contacts about potential labour exploitation in industries that remained open during restrictions unexpectedly rose, possibly indicating exploiters moved victims/survivors from restricted to essential industries, as suggested by preliminary Helpline analysis [27].

Contrary to expectations, contacts about potential in-person sexual exploitation did not decrease, nor did those about online sexual exploitation increase after restrictions were implemented (thus, H_5_ and H_6_ were not supported). The latter may reflect exploitation being harder to detect online [53]. The classification of 'online' in the data may though reflect where suspected exploitation was encountered, not where it occurred, making the line between online and offline sexual exploitation more porous than it seems. A US study using proxies for online commercial sexual services suggested fluctuating demand during the restriction period [24] but given divergent trends in UK and US Helpline data [8], these patterns may not be comparable.

### Limitations

We address the data limitations in detail elsewhere [8], but here we emphasise the volatility of Helpline data when disaggregated into exploitation subtypes. The UK Helpline covers a broader range of exploitation scenarios compared to others, such as the US Polaris Helpline. While disaggregating data is recommended [22, 54], it results in smaller sub-samples, limiting the ability to perform more sophisticated analyses. Thus, while our study provides a detailed description of trafficking-related concerns, it could not establish causal inferences. Like other studies on exploitation during the pandemic [19], we lacked data to approximate a counterfactual or the ability to control for confounders. Consequently, we cannot definitively say that the observed trends were directly caused by the restrictions. Indeed, cases of potential domestic servitude and victim/survivor self-reports have continued to rise beyond our data period [47], suggesting that the pandemic may have revealed underlying patterns rather than caused them. Furthermore, it remains unclear to what extent the belated reporting effect seen in other UK data might apply to the Helpline data used here [50], and how this may have obscured patterns.

### Implications

Helplines, due to their virtual nature, are technically less vulnerable to disruption than in-person services [55] providing there is a stable funding climate. The increased self-reporting patterns found here may reflect displacement from in-person services to the Helpline, indicating its importance as a public health intervention within the services ecosystem. In future crises, policymakers and anti-trafficking professionals should have clear coordination plans across the service infrastructure. Those should anticipate increased Helpline contacts for exploitation types/sub-types that become more visible and adequate resourcing to support victims/survivors. Other organisations better placed to identify forms of exploitation made more hidden by the crisis should also be scaled up. Supporting referral pathways from Helplines to follow-up services is crucial. And those follow-up services need to be adequately funded to provide high-quality, easy-to-access, and inclusive care to diverse service users to reduce the health and social impacts of harms caused [8].

### Future research

To plan effective service provision for trafficking victims/survivors during future crises, the mechanisms behind the patterns identified in this research must be better understood. Our conjecture about these trends’ causes require further exploration and testing. Qualitative research with affected communities is sorely needed to understand their pandemic experiences, health needs, and factors driving help-seeking, shedding light on the well-documented lag between exploitation and reporting [50]. This insight could improve service access during crises and generate theory grounded in lived experience that supports a realist public health approach [56, 57] and builds the nascent anti-trafficking evaluation evidence-base [1]. Finally, research done amid future crises should leverage Helpline data as a ‘pulse check’ of public and professional sentiment about exploitation [58], to enable the service infrastructure to swiftly adapt to service-users’ real-time needs.

### Conclusion

This study capitalised on rare access to a large, independent, dataset on contacts to the UK’s Modern Slavery & Exploitation Helpline over five-years, including the period when public health restrictions associated with Covid-19 were in place. We found a shift in the composition of contacts over the pandemic, with more potential victims/survivors seeking help, and a sharp decline in community reports of suspicions. This is grounds for celebration, as the Helpline can better advise and support contacts with direct insight into their exploitation. While overall case numbers did not rise significantly, this does not imply trafficking prevalence remained unchanged, given the socially constructed nature of Helpline data and complex systems involved [10]. But a detailed and nuanced understanding of the types of contacts and the conditions they report provide crucial information for service design and delivery. The relevance of this extends beyond pandemics into disaster planning for other scenarios that disrupt physical and social infrastructure, including natural disasters and conflicts.

## Data Availability

The data that support the findings of this study are available from Unseen, but restrictions apply to the availability of these data, which were used under licence for the current study and so are not publicly available. Because of the sensitivities of the data and the terms of the legal agreement governing their provision, the authors are unable to make the data available for onward use.

## Footnotes

1 Known in the UK as ‘County Lines’, for it involves moving drugs from urban centres to provincial county towns, often exploiting children and young people as drug couriers.

2 Stay-at-home orders generally ran from 13 March 2020 to 12 April 2021, with geographical variation across the UK [39].

3 In-person working was restricted to all but essential workers from 21 March 2020 to 1 October 2021 [39].

4 The UK government defined these sectors as: health and social care, education and childcare, key public services, local and national government, food and other necessary goods, public safety and national security, transport and border, utilities, communication and financial services.

5 From 2022 the Helpline began including labour abuse, signalling an expansion to cover abusive practices beneath the threshold to be considered ‘modern slavery’. The data used herein does not include such cases [47].

6 Indirect contact means the contact had heard about the potential victim/survivor third hand, whereas direct contact means those who had first-hand communication with them.

7 For brevity we use the term contact throughout to refer to anyone contacting the Helpline but note that most contacts were made by calling the Helpline.

8 As recorded in the Oxford COVID-19 Government Response Tracker data, [39] restrictions were lifted and re-imposed in different UK regions at different times over the ‘during pandemic’ period.

9 Unseen confirmed that most contacts recorded as ‘drugs’ relate to County Lines (personal communication, 15 May 2024).

10 Municipal government agencies.

11 Strict restrictions on gatherings were in place 23 March 2020 to 10 September 2021.
